# 6DYN : 6TiSCH with Heterogeneous Slot Durations

**DOI:** 10.3390/s21051611

**Published:** 2021-02-25

**Authors:** Mina Rady, Quentin Lampin, Dominique Barthel, Thomas Watteyne

**Affiliations:** 1 Orange Labs, 38240 Meylan, France; quentin.lampin@orange.com (Q.L.); dominique.barthel@orange.com (D.B.); 2 The National Institute for Research in Computer Science and Automation (Inria), EVA Team, 75012 Paris, France; thomas.watteyne@inria.fr

**Keywords:** 6DYN, 6TiSCH, technology agility, OpenWSN

## Abstract

New radio chips implement different physical layers, allowing firmware to change modulation, datarate and frequency dynamically. This technological development is an opportunity for industrial low-power wireless networks to offer even higher determinism, including latency predictability. This article introduces 6DYN, an extension to the IETF 6TiSCH standards-based protocol stack. In a 6DYN network, nodes switch physical layer dynamically on a link-by-link basis, in order to exploit the diversity offered by this new technology agility. To offer low latency and high network capacity, 6DYN uses heterogeneous slot durations: the length of a slot in the 6TiSCH schedule depends on the physical layer used. This article shows how reserved bits in 6TiSCH headers can be used to standardize 6DYN and details its implementation in OpenWSN, a reference implementation of 6TiSCH.

## 1. Introduction

Low-power wireless mesh networks are used in a wide range of applications which require a high level of dependability, including predictive maintenance [1], smart grid monitoring [2], building energy management [3] and precision agriculture [4]. A class of networking protocols has been developed for high reliability and low power performance based on Time Slotted Channel Hopping (TSCH). In a TSCH network, time is divided into slots, where in each slot a device may transmit, receive or sleep, and communication happens over multiple frequencies to improve link reliability.

TSCH was introduced in the 2016 IEEE802.15.4e [5] amendment to the IEEE802.15.4 standard [6]. Several complete protocol stacks build upon TSCH, including WirelessHART and ISA100.11a. Both use a central entity to build and maintain the TSCH schedule [7].

Recently, the 6TiSCH working group at the Internet Engineering Task Force (IETF) has standardized a protocol stack which combines IPv6 with the TSCH mode of IEEE802.15.4e. 6TiSCH combines wire-like reliability and ultra-low power operation, in a fully distributed approach (i.e., no central controller). It relies on O-QPSK 2.4 GHz short-range radios as defined by IEEE802.15.4 [6]. In 2015, the IEEE adopted the IEEE802.15.4g amendment [8], which included 31 new radio settings, offering a range of performance options for Smart Utility Networks (SUN). They vary in performance, from long range to high bit-rate, depending on the use-case requirements. We use the term “physical layer” (PHY) to refer to a combination of frequency band, modulation, coding scheme and symbol rate.

The diversity of the PHYs of IEEE802.15.4g helps meet the diverse requirements of industrial networks. For instance, some applications require long-range performance, which can be achieved by using lower bit-rate modulations. Other applications are more energy sensitive, in which case higher bit-rate modulations are more applicable. The traditional approach for network engineers is to compare the different radios and choose the one that best fits the network’s use case. This leads to an agility challenge: How should be addressed the case where some neighbor nodes are far apart while others are close by? This would benefit some using a longer-range PHY in the former case and a higher datarate in the latter. These cases are very commonplace in mesh networks, in which many links exist between nodes.

Recent radio chips support multiple PHYs at competitive cost and energy consumption. For instance, the Atmel AT86RF215 radio chip supports the full range of the IEEE 802.15.4g PHYs in both 2.4 GHz and sub-GHz bands [9]. The AT86RF215’s architecture allows switching between those on a frame-by-frame basis. This capability enables an agile wireless networking approach in which the PHY is chosen for each pair of neighbor nodes.

Our vision is that advanced low-power wireless networks will use agile networking to increase their performance. The 6TiSCH protocol stack, through its scheduled nature, is particularly suited to be generalized to support agile networking. This article introduces 6DYN, a 6TiSCH extension in which a node changes its PHY dynamically at each time slot, depending on which neighbor it is communicating with.

Since 6TiSCH was designed on a fixed PHY, in today’s 6TiSCH standard, all slots are of the same duration. The slot duration is tuned so there is time to transmit the longest supported frame and receive an acknowledgement. When using the IEEE802.15.4 2.4 GHz PHY at 250 kbps, a slot is typically 10 ms long. When using multiple PHYs, we can have two approaches. The first is to settle for using the longest slot duration among all PHYs. This is the approach we previously took [10]. While this makes scheduling simple, it makes most slots artificially long, wasting latency and network capacity. A more complete approach is to vary the slot duration on a slot-by-slot basis, based on the PHY used. This is the approach 6DYN uses.

This article answers the following questions:

- How can we build a 6TiSCH schedule with different slot durations?
- What are the minimal changes we need to make to the 6TiSCH standard to achieve this network agility?
- What are the benefits of 6DYN over using the longest duration?

The remainder of this article is organized as follows. Section 2 surveys related work. Section 3 states the problem and lists this article’s contributions. Section 4 presents the design of 6DYN, including time templates, slot duration management, neighbor discovery and timeslot allocation. Section 5 lists the two small changes needed to the 6TiSCH standard to support 6DYN. Section 6 describes our implementation of 6DYN in OpenWSN, the reference implementation of 6TiSCH. Finally, Section 7 concludes this article.

## 2. Related Work

This section outlines four areas of related work: 6TiSCH (Section 2.1), TSCH scheduling (Section 2.2), frequency band considerations (Section 2.3) and multi-PHY approaches (Section 2.4).

### 2.1. 6TiSCH

The 6TiSCH protocol stack [7,11] combines the ease of use of IPv6 with the industrial performance of TSCH. 6TiSCH uses IEEE802.15.4 O-QPSK 2.4 GHz as its PHY [6] and IEEE802.15.4e [5] at the Medium Access Control (MAC) layer. A 6TiSCH schedule is a matrix of cells, each identified by its slot offset and channel offset. There are three types of cells:

- A *dedicated* cell is negotiated between neighbor nodes. A TX cell is allocated at the source mote and an RX cell is allocated at the destination motes.
- A *broadcast* cell (also called “Minimal cell”) is used for network-wide broadcast messages and network routing information. This allows a mote to synchronize to the network, discover its neighbors and the network routing details.
- Each node allocates an *autonomous* cell in its schedule where it can receive negotiation requests for cell allocations from its neighbors.

6TiSCH defines an operational sub-layer (6top) which coordinates a mote’s negotiations with its neighbors to allocate cells [12]. It further defines a default scheduling algorithm, called Minimal Scheduling Function (MSF) [13], which defines how cells are allocated for unicast or broadcast activities. 6TiSCH uses the 6LoWPAN adaptation layer [14] to transport IPv6 packets over IEEE 802.15.4e MAC layer, and the IPv6 Routing Protocol for Low-Power and Lossy Networks (RPL) [15] routing protocol. Chang et al. provided a comprehensive overview of the 6TiSCH protocol stack [16].

Schedule management can be done in a centralized or distributed manner. In a centralized approach [17,18], a central entity manages the schedule based on a complete view of the network. In a distributed approach, nodes manage their resources locally. 6TiSCH uses the latter.

### 2.2. TSCH Scheduling

Hermeto et al. focused on scheduling in IEEE802.15.4-TSCH industrial networks, surveying both centralized and distributed approaches [19]. They put a particular focus on schedule compactness, indicating that “the number of channel offsets and timeslots not used by any transmitter must be maximized. A compact schedule may be convenient to admit later new flows: enough space is available to allocate more bandwidth”. They indicated that research on improving schedule compactness has been done exclusively for centralized networks. In this current paper, we focus on schedule compactness in a distributed TSCH network.

Palattella et al. proposed a Traffic Aware Scheduling Algorithm ( TASA) for schedule optimization [20]. TASA relies on a central manager to reserve slots as early as possible in the schedule. It relies on minimizing the maximum offset of used slots in the schedule. The authors ported TASA to the 6TiSCH architecture in combination with the IEEE802.15.4e MAC and provided a simulation of its performance [21]. They showed that TASA-based IEEE802.15.4e MAC shows 80% improvement in the power efficiency compared to the legacy IEEE802.15.4 MAC.

Soua et al. proposed MODESA, a centralized slot allocation algorithm which relies on a root node having multiple radio interfaces [22]. MODESA reduces the slotframe length by optimizing slot and channel allocation across a tree-topology network. The authors provided a linear programming model that runs on the central controller and optimizes slot and channel assignment. They showed how this approach reduces slotframe length by 13% in a 100-node network.

All related work surveyed in this section is based on a centralized controller, and it all assumes the same PHY is used across the network (hence, a uniform slot duration). This article proposes 6DYN, a distributed mechanism for increasing schedule compactness combined with a multi-PHY approach and heterogeneous slot durations. 6DYN allows nodes to allocate shorter slots for faster transmissions and longer slots when necessary for longer transmissions. This allows for better packing of the schedule, which reduces latency and increases network capacity.

### 2.3. Frequency Band Considerations

There are two main considerations to take into account related to the frequency band used: co-existence with other technologies in unlicensed band and duty cycle limits on sub-GHz unlicensed bands.

Hermeto et al. indicated that the articles they surveyed [20,21,22,23] do not consider packet loss due to interference from IEEE802.11 WiFi networks [19]. Since the IEEE802.15.4 PHY operates using O-QPSK 2.4 GHz, it has classically suffered interference from co-existing Wi-Fi networks. Musaloiu et al. showed how IEEE802.15.4 networks exhibit packet losses ranging 22–58% when WiFi networks are operating in the same area [24]. Gonga et al. showed that up to 95% of the links in a IEEE802.15.4 channel hopping network can suffer independent packet losses due to WiFi interference [25]. Furthermore, Liu et al. proposed an Industrial Internet of Things (IIoT) architecture that integrates satellite IIoT with ground cellular IIoT to extend coverage in satellite-blocked areas. This integration leads to co-channel interference from both networks operating on the same frequencies. The authors therefore proposed a mechanism to optimize the satellite power resource allocation to mitigate the interference and guarantee a quality of service level [26].

In Europe, the European Telecommunications Standards Institute (ETSI) is the regulatory body which governs frequency bands. Section 7.2.3 of ETSI 300-220 [27] limits the transmit duty cycle (the portion of the time a radio is actively transmitting) in the 868 MHz band between 0.1% and 1% depending on the sub-band. Similar regulation is in place in the US (under the guidance of the FCC) and other parts of the world. Such regulations must be taken into account by scheduling policy using these frequencies.

6DYN uses both the 2.4 GHz and sub-GHz bands. By increasing the available frequency resources, 6DYN reduces the probability of interference from co-existing networks. Furthermore, it reduces the impact of the duty-cycle regulation by splitting the amount of traffic between the two bands.

### 2.4. Multi-PHY Approaches

The IEEEE 802.5.4g amendment (2012) standardized new PHYs for Smart Utility Networks (SUNs) [8]. In our previous research, we evaluated the performance of the 6TiSCH protocol stack when using three of those PHYs [28]: Frequency Shift Keying (FSK) in the sub-GHz band at 50 kbps, Orthogonal Frequency Division Multiplexing (OFDM) Option 1 with Modulation and Coding Scheme (MCS) 3 in the sub-GHz band at 800 kbps and Offset Quadrature Phase Shift Keying (O-QPSK) in the 2.4 GHz band at 250 kbps. We evaluated the network performance according to industrial Key Performance Indicators (KPIs) [29] and explored the trade-offs between these PHYs. For instance, FSK 868 MHz offers 100% end-to-end reliability while degrading the battery lifetime by nearly 75% compared to O-QPSK 2.4 GHz. This study motivates integrating the PHYs in one general architecture that allows a node to change its neighboring PHY depending on link quality.

Brachmann et al. took a first step in integrating different PHYs under the 6TiSCH protocol stack [30]. They used 2-GFSK PHY at 1.2 kbps to transmit broadcast beacons for network synchronization and neighbor discovery and 4-GFSK at 1000 kbps for data transmissions. They demonstrated a multi-template slot design to integrate both PHYs in the TSCH slotframe. Use of higher bit-rate for the data transmissions reduces the channel duty-cycle to 0.1% compared to 2% for the beacon transmissions on the slower bit-rate.

Our recent work introduces g6TiSCH, a generalized 6TiSCH architecture where nodes support multiple PHYs and each node can change PHYs with their neighbors depending on link quality [10]. g6TiSCH focuses on three PHYS: FSK 868 MHz, OFDM 868 MHz and O-QPSK 2.4 GHz. We showed that g6TiSCH offers the same reliability of an FSK 868 MHz network, yet with only 20% of the links used in FSK 868 MHz. g6TiSCH does use a unique slot duration of 40 ms for all PHYs, even though slots for OFDM 868 MHz and O-QPSK 2.4 GHz do not need to be longer than 10 and 20 ms, respectively.

6DYN builds upon the related work surveyed in this section and adds heterogeneous slots durations: nodes dynamically negotiate chains of slots of variable lengths, depending on the PHY used.

## 3. Problem Statement and Contributions

In their IETF problem statement, Muñoz et al. discussed the challenges of integrating heterogeneous PHYs in 6TiSCH [31], including multi-PHY discovery of neighbors and routing.

**Figure 1:**
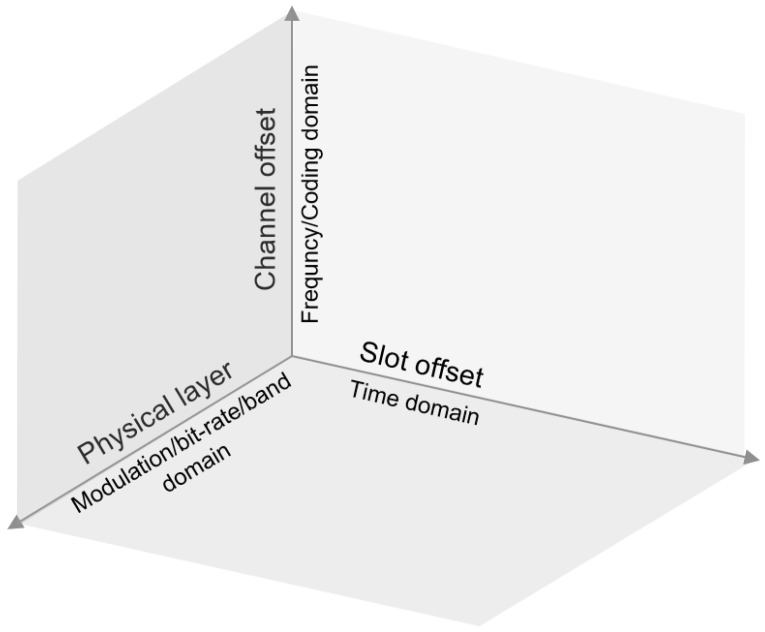
6DYN assigns a PHY to each communication opportunity, on top of a timeslot and a channel offset.

6DYN is one answer to those challenges, with a particular focus on schedule compactness. In a 6TiSCH network today, a communication opportunity between neighbor nodes is identified by a timeslot and a channel offset. 6DYN adds one dimension to this, the PHY, as illustrated in Figure 1. The result is that, when neighbor nodes communicate, they previously agree on the frequency band, modulation and bit-rate that make their communication most efficient.

The contribution of this article is three-fold:

1. 6DYN, a TSCH approach in which nodes use multiple PHYs in an efficient manner by managing a communication schedule with multiple slot durations
2. A draft standardization of 6DYN by crafting it as an extension of 6TiSCH
3. An implementation of 6DYN in OpenWSN, a reference open-source implementation of 6TiSCH

## 4. 6DYN: A TSCH Network with Heterogeneous Slot Durations

**Figure 2:**
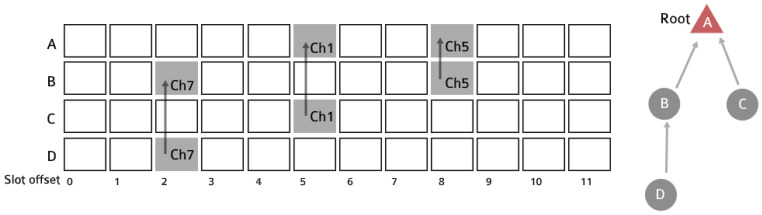
A typical TSCH slotframe for Motes A–D, using a uniform slot duration and single physical layer at a fixed bitrate. Each node allocates a specific slot offset and channel offset for transmissions to its parent.

A typical TSCH slotframe is depicted in Figure 2. In this example, each node in the network allocates a dedicated cell for its parent for up-link traffic. All nodes in the network use a uniform slot duration to construct their schedules, since all nodes only use a single PHY, O-QPSK 2.4 GHz at 250 kbps. In IEEE802.15.4, which 6TiSCH is based on, the maximum MAC payload is 127 B, which takes 4 ms to be transmitted. Taking into account the time-on-air of both the data frame and the acknowledgement that follows, as well as the turn-around and processing times, a slot duration of 10 or 20 ms is typical.

With the integration of the IEEE802.15.4g amendment, the IEEE802.15.4 standard comprises a wide range of PHYs. At sub-GHz, the PHY with the extreme bitrates are FSK 868 MHz at 50 kbps and OFDM 868 MHz at 800 kbps. At 2.4 GHz, O-QPSK 2.4 GHz offers a middle ground at 250 kbps, on a different frequency band. We showed that these three PHYs are complementary to one another, and being able to dynamically switch between them on a link-by-link basis offers diversity, which increases network performance [28]. Table 1 lists the PHYs [9,32].

Our previous proposal, g6TiSCH [10], uses those three PHYs, but with a uniform slot duration, 40 ms. 6DYN is an extension of g6TiSCH where slots have different durations, as a mechanism to lower latency and increase network capacity.

This section introduces 6DYN. Section 4.1 focuses on the timeslot templates for each PHY. Section 4.2 describes how slots with different durations can be combined in a single schedule. Section 4.3 details how neighbor nodes discover one another, on each PHY. Section 4.4 details how cells of different durations are allocated.

### 4.1. Timeslot Templates

To support each PHY in the same schedule, we first need to design their timeslot templates in accordance with IEEE 802.15.4e standard. A timeslot template consists of the different times within a timeslot when the node must turn its radio on/off, transmit and receive, as well as the total duration of that slot. A slot must be long enough for neighbor nodes to exchange the longest frame and the subsequent acknowledgement. Its duration hence depends on the length of the frame, the bit rate of the PHY, and any action internal to the mote that takes time (executing code, transferring data over an SPI bus, etc.).

**Figure 3:**
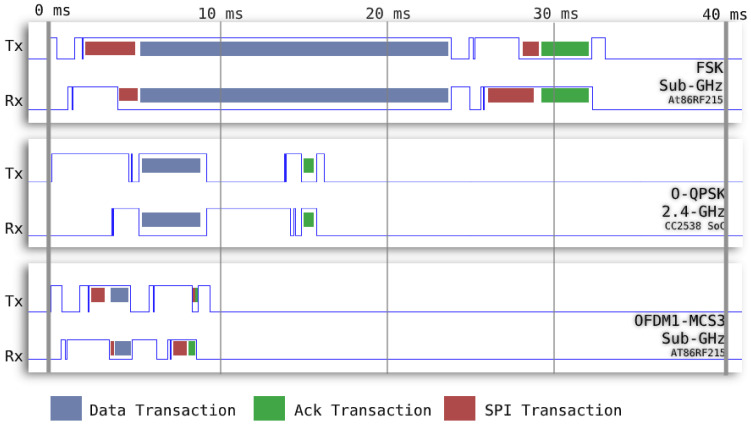
Logic analyzer capture of the custom timeslot templates for each PHY in accordance with the IEEE802.15.4e standard.

We tune the timeslot template experimentally by placing two OpenMote B boards side by side and programming them to exchange a frame and acknowledgement for each of the PHYs. We instrument the code to toggle pins at particular times in the state machine and use a logic analyzer to visualize that activity. Figure 3 is a capture of the logic analyzer, showing when the motes exchange a 127 B data frame (the maximum length), exchange an acknowledgement frame and communicate over SPI. We “push” the activity as much to the left as possible, so that the interaction is over as fast a possible within the slot.

We note that the FSK 868 MHz and OFDM 868 MHz PHYs require time for SPI transactions between the radio chip (AT86RF215) and the micro-controller (CC2538). For O-QPSK 2.4 GHz, the radio front-end is part of the CC2538 in a system-on-chip design. Figure 3 shows that OFDM 868 MHz, O-QPSK 2.4 GHz and FSK 868 MHz transactions are over in roughly 10, 20 and 40 ms, respectively. We choose these “round” numbers because they make the slot lengths multiples of one another, significantly simplifying scheduling (see Section 4.2).

### 4.2. Heterogeneous Slot Durations

How then can the different slot durations be integrated in the same slotframe? The approach by Brachmann et al. [30], inspired by IEEE802.11 WiFi networks, is to use the slowest bit rate PHY with a specific slot duration for control packets, and the highest bitrate PHY for data packets and grouping multiple slots for it. While this approach improves schedule compactness, it does not give the node the flexibility to use lower bit-rates for data transmissions in case the quality is poor.

Another approach in g6TiSCH [10] is to use a uniform slot duration based on the slowest PHY (40 ms) while allowing the node to switch between the three PHYs for data packet depending on link quality. This gives the mote flexibility to tune its performance by switching to faster bit-rates when possible or to slower bit-rates when necessary. It is also easier to manage the schedule using the existing scheduling policies that are designed to manage slots with a uniform slot duration. This does lead to schedule compactness and capacity problems: in each OFDM 868 MHz and O-QPSK 2.4 GHz slot, the mote wastes 30 or 20 ms, respectively.

In this article, we extend the approach of g6TiSCH by allowing for slots of different durations. We set the slot duration to that of the *fastest* PHY, 10 ms, and *group consecutive slots* when using slower PHYs, resulting in some longer “virtual” slots. Given the durations listed in Section 4.1, an O-QPSK 2.4 GHz slot occupies two consecutive slots, while an FSK 868 MHz slot occupies four consecutive slots. We use this approach for any dedicated cell that is dynamically negotiated between neighbors and use the slowest bit-rate (FSK 868 MHz ) for autonomous cells. Autonomous cells are used to “bootstrap” the negotiation.

**Figure 4:**
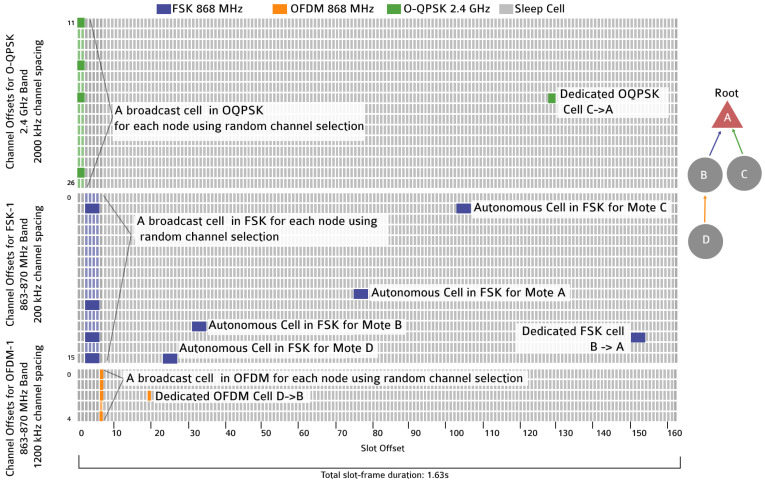
A heterogeneous slotframe for an example multihop topology, including broadcast cells for multi-PHY network discovery.

Figure 4 depicts the resulting heterogeneous slotframe for a multi-hop network of three motes and one root. On top of the 16 frequencies for O-QPSK 2.4 GHz, this schedule also includes 16 FSK 868 MHz frequencies with 200 kHz channel spacing and 5 OFDM 868 MHz frequencies with 1.2 MHz channel spacing. We use a slotframe length of 163 timeslots, resulting in a duration of 1.63 s, comparable to the 1.64 s used in g6TiSCH. A separate minimal cell is allocated at the beginning of the slotframe for each of the PHYs, for network-wide communication. Autonomous cells are allocated using the longest range FSK 868 MHz PHY.

### 4.3. Neighbor Discovery

Given that 6TiSCH is a multi-hop mesh network, nodes need to discover one another, on different PHYs. Among other things, this allows each node to pick the most suitable neighbor and the most suitable PHY, for up-link communication. In traditional 6TiSCH, all nodes that are part of a network send enhanced beacons on the schedule’s minimal cell at a slow rate. By listening on that cell when not sending, a node eventually builds up a list of all the other nodes it can hear, i.e., its neighbors.

We adapt this discovery mechanism to multi-PHY discovery by having three distinct minimal cells, one for each PHY. Figure 4 shows these cells. If a node has not joined the network yet, it listens for beacons using its FSK 868 MHz radio. After it hears an enhanced beacon, the node synchronizes to the network and continues listening for enhanced beacons only on the minimal cell. A mote’s neighbor table contains one row per tuple (MAC address, PHY). This allows keeping the upper layers unchanged, notably the routing layer.

This decision finds a balance between two concerns. First, the changes in PHY and MAC layers do not impact any of the routing protocol dynamics. This allows seamless integration into the routing layer; the agility of the 6TiSCH protocol stack is maintained. Second, each PHY for a given neighbor is stored in a different row in the neighbor table. This trick causes the routing to treat different PHYs as different neighbors. It automatically takes the cost associated with each PHY into account and switches between high bit-rate and low bit-rate PHYs depending on link quality. The network dynamically improves its power consumption over time.

### 4.4. Timeslot Allocation

In a distributed network, neighbor nodes negotiate with one another to add/delete dedicated cells in their schedule to communicate. This mechanism is augmented to support multi-PHY operation: schedule a single cell to add an OFDM 868 MHz cell, schedule two consecutive cells to add an O-QPSK 2.4 GHz cell and schedule four consecutive cells to add an FSK 868 MHz cell. The node issuing the transaction proposes groups of contiguous cells that are unused in its schedule; the other node selects a group it is not using itself.

## 5. Extending 6TiSCH with 6DYN

As a path towards standardization, this section details how the 6TiSCH standard can be amended to support 6DYN. The goal is to make only small adjustments to the standard to simplify implementation and favor adoption.

**Figure 5:**
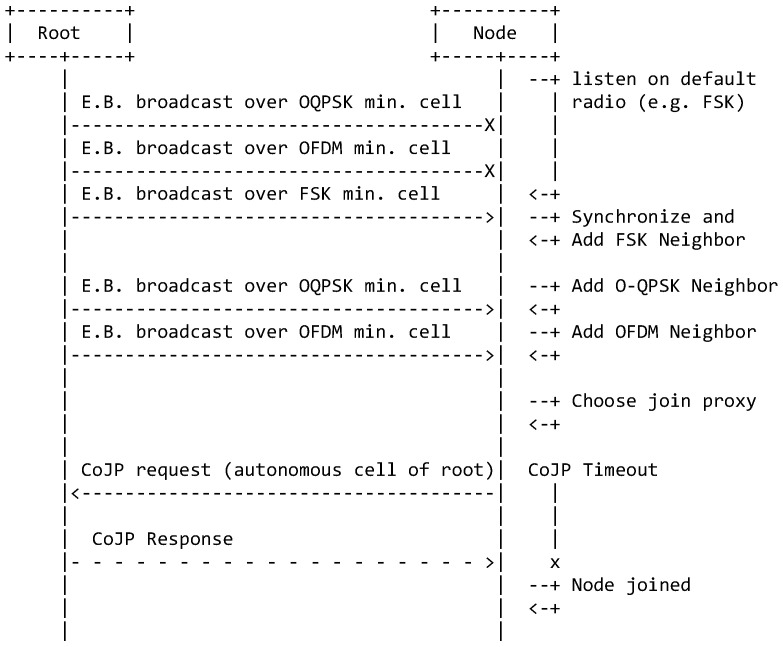
Synchronization and join process when using 6DYN.

The first change is to encode the PHYs used in the network in the Enhanced Beacon (EB) [33]. Per the IEEE802.15.4 standard, the EB encodes the slot offsets of each minimal cell in the TSCH slot frame, and Link Information Elements [33]. In the Link Information Elements for each minimal cell, we use the left-most 3 bits of the Link Options byte to encode the type of PHY associated with each minimal cell. These bits are currently “reserved for future use” in the IEEE802.15.4 standard. This allows a mote that hears an EB during the joining process to bootstrap its schedule correctly. This mechanism further allows a network to use only two of the PHYs listed in Table 1. The remaining steps of the secure joining procedure of a node is carried out by the Constrained Join Protocol (CoJP) [34] and is unmodified. The resulting join process is illustrated in Figure 5.

**Figure 6:**
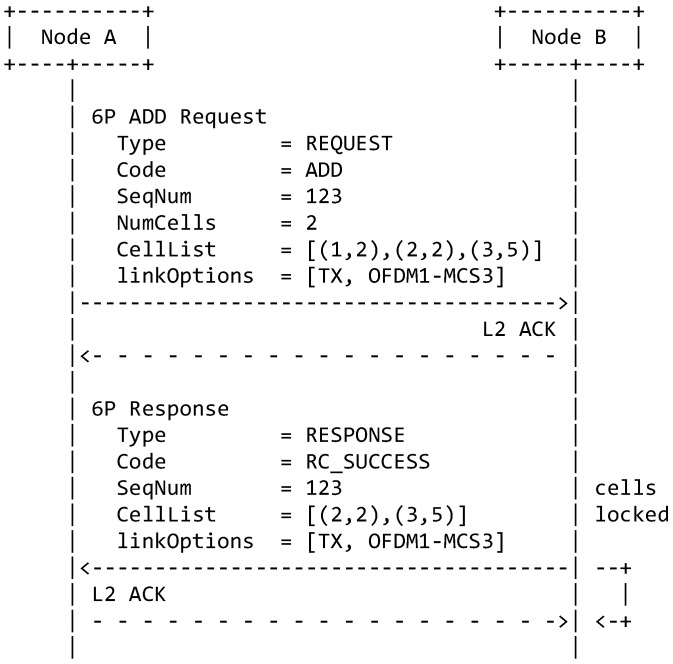
A 6P ADD transaction when using 6DYN.

The 6top Protocol (6P) is used by neighbor nodes to negotiate when adding/deleting cells with one another [12]. The second change is to extend 6P for an ADD request to indicate which PHY to use. We use the left-most 3 bits of the Cell Options byte in the 6P header to encode the PHY index of the requested cells. These bits are currently “reserved for future use” in the 6P standard. The resulting 6P ADD transaction is illustrated in Figure 6.

These two changes, while requiring a rigorous definition through an official standard, remain small changes to the standard. The remaining of the 6TiSCH standard remains unmodified.

## 6. Implementing 6DYN in OpenWSN

OpenWSN is the reference implementation of 6TiSCH, used for example during 6TiSCH interoperability tests [35]. We extend OpenWSN with 6DYN. Section 6.1 details the hardware used, include the debugging infrastructure. Section 6.2 highlights the key elements in the implementation of 6DYN. Section 6.3 shows 6DYN running on the OpenMote B board.

### 6.1. Experimental Setup

**Figure 7:**
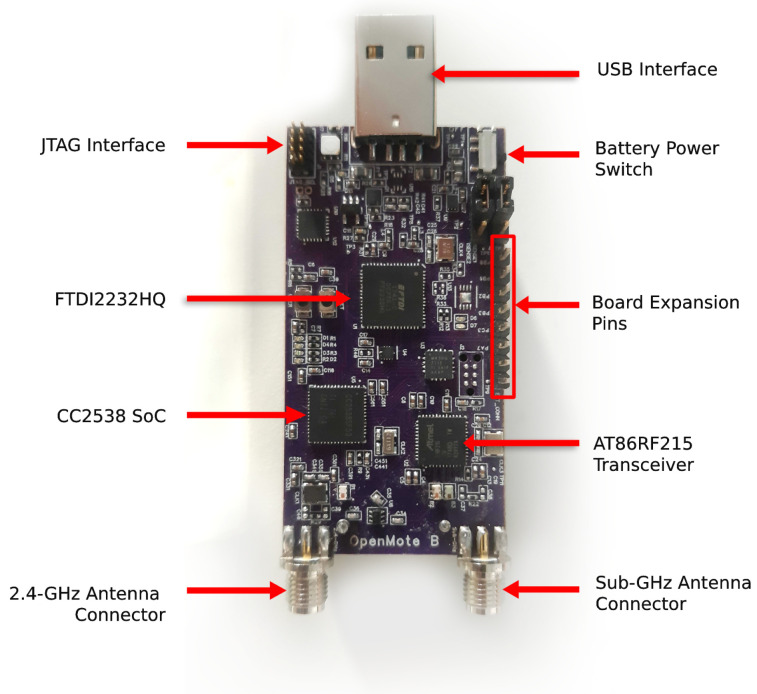
The OpenMote B board, used for running 6DYN.

We use the OpenMote B (Figure 7) as our development platform. It features the CC2538 SoC as well as the AT86RF215 radio, implementing all PHYs listed in Table 1. We use a JTAG interface for in-circuit debugging and the IAR Embedded Workbench as the Integrated Development Environment.

The OpenMote B routes six general purpose input/output pins from the CC2538 to extension headers, which we use to follow the execution of the firmware using a logic analyzer. We instrument the firmware to toggle the following pins:

- The slot pin is toggled at the start of each timeslot.
- The radio is set high when the radio is on, either in transmit or receive mode.
- The fsm pin toggles at each transition of the Finite State Machine of the implementation of the TSCH MAC layer. This pin allows us to identify transmit and receive slots and the timing inside the slot, defined in the timeslot template (see Section 4.1).

### 6.2. Implementing 6DYN

We implemented 6DYN in OpenWSN (as an online addition to this article, all of the source code used is available at https://github.com/openwsn-berkeley/ (accessed on 24 February 2021)) according to the architecture indicated in Section 4 and the frame defined in Section 5. The implementation depends on the openradio interface, a PHY-layer agile extension to OpenWSN [28]. While the implementation is relatively straightforward, we believe the following details to be particularly important.

In our implementation, we encode the communication schedule as a circular linked list of cells, in increasing slot offset order. We only use a single entry in that linked list to represent a cell, regardless of the PHY used. This means that an FSK 868 MHz timeslot, which spans 40 ms (i.e., four cells), occupies the same memory footprint as a 10 ms OFDM 868 MHz timeslot. The fact that timeslots are ordered in a circular linked list allows the implementation to iterate to the next slot without a time-consuming search operation.

The default implementation of MSF in OpenWSN allocates multiple entries in the same timeslot (i.e., backup slots). This is done so that, if there are no packets associated with this timeslot, the MAC layer executes a lower-priority slot entry [13,36]. This implementation does not carry over directly to 6DYN, as slots can be partially overlapping. In our implementation, backup slots are allowed only for slots of the same size.

The flash memory footprint of g6TiSCH without the 6DYN extension is 79 kB. Implementing 6DYN increases that footprint to 84 kB, still very far from filling up the 512 kB of flash memory available on the OpenMote B.

### 6.3. Running 6DYN

To “witness” the execution of 6DYN, we use OpenMote B boards to form a link between two motes and a multi-hop network of four motes. On top of the 6DYN-enabled 6TiSCH protocol stack, on each mote, we run an application which periodically reports an incrementing counter (used to compute end-to-end reliability) and the radio duty cycle of each PHYs.

**Figure 8:**
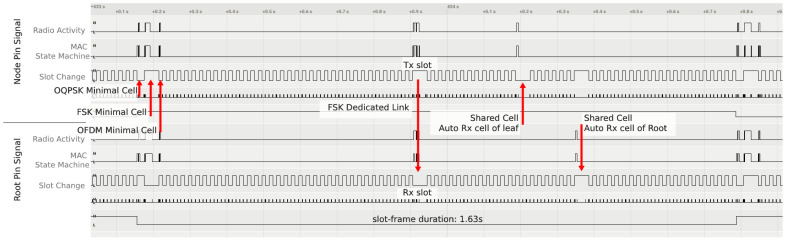
Logic analyzer trace showing the activity of two neighbors nodes communicating using 6DYN.

Figure 8 shows the activity of two neighbor nodes, as captured by a logic analyzer connected to both. Slot are 10 ms long, the slotframe is 163 slot long. On the left, per the neighbor discovery detailed in Section 5, we see the three minimal cells: a 20 ms O-QPSK 2.4 GHz cell, a 40 ms FSK 868 MHz cell and a 10 ms OFDM 868 MHz cell. Figure 8 shows a dedicated cell using FSK 868 MHz, which the nodes negotiated using 6P, as detailed in Section 5. Finally, we see each node switching on its radio during its autonomous cell; each node listens for a short period and turns off its radio as no frame is received.

**Figure 9:**
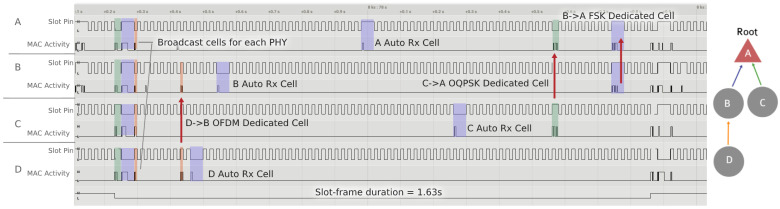
Logic analyzer trace showing the activity of four motes implementing 6DYN and forming a multi-hop network. The resulting schedule corresponds to Figure 4.

Figure 9 shows the activity of four motes implementing 6DYN and forming a multihop network. It shows how different pairs of nodes establish different links (D→B, B→A and C→A), incidentally each using a different PHY.

## 7. Conclusions

New radio chips implement multiple PHY layers, offering different trade-offs among range, data rate and power consumption. Because a low-power wireless network is composed of many links, each with different characteristics, being able to dynamically change between PHYs on a link-by-link basis yields higher network performance than locking the entire network to a single PHY. Recent proposals, such as a g6TiSCH, propose a networking approach which allows for this technology agility, in a timeslotted and scheduled manner. They do so, however, by aligning the duration of a timeslot to the slowest PHY layer. While this simplifies the implementation, the time “wasted” when using faster PHYs results in increase latency and reduces network capacity.

This article introduces 6DYN, a time-synchronized channel hopping protocol which uses heterogeneous timeslot durations. 6DYN defines different timeslot durations: 10 ms when using OFDM 868 MHz, 20 ms when using O-QPSK 2.4 GHz and 40 ms when using FSK 868 MHz. 6DYN offers diversity across modulations, data rate and frequency bands. We detail how 6DYN achieves multi-PHY neighbor discovery and how timeslots are allocated. We also show how reusing two sets of reserved bits in packet headers makes 6DYN easy to standardize as an extension to 6TiSCH. We implemented 6DYN in OpenWSN, the reference implementation of 6TiSCH, an extension that is available to the community.

We are currently working on an exhaustive performance evaluation of 6DYN, compared to 6TiSCH and g6TiSCH, using the OpenTestbed. We are particularly interested in measuring the reduction of latency and the increase of the overall determinism of the network by this multi-PHY approach. We are also interested in how routing metrics can be adapted to improve the overall network energy footprint for the given PHYs used in the network. We see 6DYN as a step towards deterministic networks in industrial applications (e.g., control loops), made possible by multi-PHY chips.

## Figures and Tables

**Table 1:** The PHYs used by 6DYN.

	FSK 868 MHz	OFDM 868 MHz	O-QPSK 2.4 GHz
radio chip	AT86RF215	AT86RF215	CC2538
frequency band	sub-GHz	sub-GHz	2.4 GHz
data rate	50 kbps	800 kbps	250 kbps
output power	+14.5 dBm	+10.0 dBm	+7.0 dBm
sensitivity	−114 dBm	−104 dBm	−97 dBm
link budget	128.5 dB	114.0 dB	104.0 dB
